# Barriers and Facilitators to Healthcare Service Access among Persons with Spinal Cord Injury (SCI)

**DOI:** 10.1093/eurpub/ckac129.475

**Published:** 2022-10-25

**Authors:** O Bychkovska, C Egen, V Strøm, A Juocevicius, P Tederko, M Arora, L Rizzo Battistella, JP Engkasan, A Gemperli

**Affiliations:** 1 Health Service Research Unit, Swiss Paraplegic Research, University of Lucerne, Nottwil, Switzerland; 2 Department of Health Sciences and Medicine, University of Lucerne, Lucerne, Switzerland; 3 Swiss School of Public Health, Zurich, Switzerland; 4 Department of Rehabilitation, The Hannover Medical School, Hannover, Germany; 5 Research Department, Sunnaas Rehabilitation Hospital, Alværn, Norway; 6 Department of Rehabilitation, Vilnius University, Vilnius, Lithuania; 7 Department of Rehabilitation, Medical University of Warsaw, Warsaw, Poland; 8 Rehabilitation Department, University of Sydney, Sydney, Australia; 9 Department of Physical and Rehab Medicine, University of São Paulo, São Paulo, Brazil; 10 Department of Rehabilitation Medicine, Universiti of Malaya, Malaya, Malaysia; 11 Center for Primary and Community Care, University of Lucerne, Lucerne, Switzerland

## Abstract

**Background:**

Despite having a high healthcare need, persons with complex conditions are less likely to receive comprehensive care. Individuals with SCI experience difficulties accessing services 2-4 times more than the general population. There is little agreement concerning the factors that influence these access restrictions. Few studies focus on health system impact on characteristics on access.

**Objective:**

To outline barriers and facilitators to service access among persons with SCI across 22 countries in terms of health system characteristics

**Methods:**

InSCI(2017): 1st community survey on experience of persons with SCI, 12591 participants, 22 countries (Australia, Brazil, China, France, Germany, Greece, Indonesia, Italy, Japan, Lithuania, Malaysia, Morocco, the Netherlands, Norway, Poland, Romania, South Africa, South Korea, Spain, Switzerland, Thailand, USA).

**Data analysis:**

1. Hierarchical cluster analysis based on Gower distance (to group systems by access restrictions: Acceptability, Approachability, Availability, Affordability, Appropriateness).

2. Generalized linear mixed-effects decision tree (to explore the association of system characteristics and access, including WHO and OECD system indicators (e.g. UHC index, expenditure, human resources). Missing values were imputed with missforest.

**Results:**

12% of persons with SCI reported having an access restriction, most of them (7%) with Availability. By country, the highest unmet needs were reported in Poland (25%), Germany, Lithuania, and Romania (13).

1. Cluster analysis: 7 health systems clusters (groups) were identified.

2. By June 2022, we will have the results of the second analysis: the association of system characteristics with access and how it is modified by socio-demographic and medical factors.

**Expected conclusions:**

The study identifies factors a country could modify in order to improve access and strengthen the system for persons with SCI/disability, that might be relevant to general population as well.

**Key messages:**

• Persons with SCI often experience similar access restrictions across countries, incl. those with high-performing health systems. System strengthening in this area is further required in all countries.

• Health systems are fragmented, e.g. healthcare quality and access inside a country differs by region, urban/rural setting etc., hence, the systems are challenging to classify.

